# A systematic review with epidemiological update of male genital schistosomiasis (MGS): A call for integrated case management across the health system in sub-Saharan Africa

**DOI:** 10.1016/j.parepi.2018.e00077

**Published:** 2018-11-23

**Authors:** Sekeleghe Kayuni, Fanuel Lampiao, Peter Makaula, Lazarus Juziwelo, E. James Lacourse, Jutta Reinhard-Rupp, Peter D.C. Leutscher, J. Russell Stothard

**Affiliations:** a Department of Parasitology, Liverpool School of Tropical Medicine, Pembroke Place, Liverpool L3 5QA, United Kingdom; b MASM Medi Clinics Limited, Medical Aid Society of Malawi (MASM), MASM House, 22 Lower Scalter road, P.O. Box 1254, Blantyre, Malawi; c College of Medicine, University of Malawi, Private Bag 360, Chichiri, Blantyre 3, Malawi; d Research for Health, Environment and Development (RHED), P.O. Box 345, Mangochi; e National Schistosomiasis and STH Control Program, CHSU, Ministry of Health, Lilongwe. Malawi; f Merck Global Health Institute, Ares Trading S.A., Route de Crassier 15, 1262 Eysins, Switzerland.; g Centre for Clinical Research, North Denmark Regional Hospital & Department of Clinical Medicine, Aalborg University, Region Nordjylland, Denmark

**Keywords:** Male genital schistosomiasis, Urogenital schistosomiasis, Praziquantel, HIV, Control

## Abstract

Male genital schistosomiasis (MGS) is a gender specific manifestation of urogenital schistosomiasis (UGS) first described in 1911 by Madden in Egypt. Today, while affecting millions of men and boys worldwide, MGS receives insufficient attention, especially in sub-Saharan Africa (SSA). To provide a systematic review with an epidemiological update of MGS, we inspected both online and hardcopy resources in our appraisal. A total of 147 articles were eventually identified, only 31 articles were exclusively focused on MGS with original or targeted research. From these, we discuss pertinent clinico-pathological features of MGS, highlight the possible connection and interplay with HIV, and assess current diagnostic techniques alongside consideration of their use and application in SSA. To appreciate the burden of MGS more fully, especially in endemic areas, there is a clear need for better surveillance and longitudinal population research to investigate the best point-of-care (POC) diagnostic and its performance through time. Furthermore, to optimise individual case management, exploration of alternative praziquantel dosing regimens is needed for MGS in men with or without HIV co-infection.

## 1 Introduction

Schistosomiasis is a snail-borne disease of humans caused by parasitic helminths of the genus *Schistosoma* (Colley et al., 2014). It remains a major neglected tropical disease (NTD) and a significant public health challenge in low and middle-income countries (Chitsulo et al., 2000; Engels et al., 2002; Christinet et al., 2016). There it causes significant morbidity and in certain areas mortality (van der Werf et al., 2003), however, the burden of schistosomiasis is underestimated due to incomplete disease surveillance as undertaken by often stretched national healthcare systems and national control programmes (King et al., 2005; Gryseels et al., 2006). The latter is more focused on tracking the delivery and treatment coverage of mass treatment campaigns offering donated praziquantel (PZQ), typically to school-aged children (Savioli et al., 2017) rather than monitoring the disease in adults per se. The consequences and disability caused by gender specific manifestations of urogenital schistosomiasis (UGS) in adults often go unremarked at national and local levels. In contrast to female genital schistosomiasis (FGS), male genital schistosomiasis (MGS), as evidenced by schistosome eggs (usually those of *Schistosoma haematobium*) in male genital organs and reproductive tracts thereof, remains poorly reported, much understudied and often misunderstood. This review was conducted to draw attention to the current evidence on MGS and assess its public health importance across the world.

### 1.1 A brief history of schistosomiasis

Corroborated references to signs and symptoms ascribed to UGS can be traced back to 1900 BCE since haematuria (i.e. frank blood in urine) was described as a common occurrence and linked to ‘menstruation’ in Egyptian males (Davis and Ansari, 1973). Schistosomiasis is a proven disease of antiquity for *S*. *haematobium* ova have been found in kidney tubules of two Egyptian mummies from 1250 to 1000 BCE (Ruffer, 1910) and more recently *Schistosoma japonicum* ova retrieved within Chinese cadavers dated to 206 to 220 CE (Coon, 2005). Schistosomiasis itself was originally described in Egypt by the German pathologist Theodor Bilharz in 1851 who discovered male and female schistosome worms at autopsy, naming them all *Distomum haematobium*. This led him to ascribe, incorrectly, that UGS and hepato-intestinal disease were linked to this schistosome species alone (Rollinson, 2009). Some sixty years later, and again in Egypt, this unfortunate mistake and subsequent confusion was fully resolved by Robert T. Leiper who demonstrated the independent lifecycles of *S*. *haematobium* and *Schistosoma mansoni* (Leiper, 1916) and their respectively aetiology in urinary and hepato-intestinal disease (Leiper, 1916; Stothard et al., 2016).

Out of the 24 species of schistosomes recognised worldwide, only six cause human diseases, namely *S*. *haematobium*, *S*. *mansoni*, *S*. *japonicum*, *Schistosoma mekongi*, *Schistosoma intercalatum* and *Schistosoma guineensis* (Rollinson, 2009). The first three species are the most important from a public health perspective. Although there may be exceptions owing to ectopic egg laying sites, *S*. *haematobium* is exclusively associated with UGS which is widely distributed in Africa and adjacent regions, affecting more people (112 million) than all other species [(WHO) see http://www.who.int/schistosomiasis/epidemiology/table/en/]. *Schistosoma mansoni*, *S*. *japonicum* and the other species causes hepato-intestinal schistosomiasis, with *S*. *mansoni* prevalent in the Caribbean, South America and Africa and *S*. *japonicum* in Asia as South East Asia (Colley et al., 2014). Of note, *S*. *mansoni*, *S*. *japonicum*, *S*. *intercalatum* and *S*. *guineensis* have been reported to cause genital manifestations but even collectively can be considered as minor when compared against *S*. *haematobium* alone.

### 1.2 Focus on male genital schistosomiasis

Male genital schistosomiasis is a specific manifestation of schistosomal disease, associated with presence of ova and pathologies thereof in various genital organs and reproductive fluids. The original report of MGS was made by Professor Frank Cole Madden, Professor of Surgery at Kasr-el-Ainy Hospital in Cairo, Egypt. In 1911, he described a 14-year Egyptian boy having enlarged scrotum showing epidydimal schistosomiasis and an English soldier complaining of haemospermia (blood in semen) concurrently with urinary schistosomiasis (Madden, 1911).

Other symptoms of MGS described in literature include pelvic pain appearing spontaneously, during coitus or on ejaculation, ejaculate changes, erection discomfort or dysfunction, infertility (Mabey et al., 2013; Farrar et al., 2014; Squire and Stothard, 2014). Although observations indicate that genital organs are frequently infested with schistosome eggs along with the urinary bladder (*S*. *haematobium*) or intestines (*S*. *mansoni*), the current extent of morbidity associated with MGS in endemic areas remains under-researched but is most clearly evidenced by post-mortem studies and case reports. By contrast, as colposcopy is available for diagnosis, ongoing surveillance of FGS has been better reported particularly in light of its three-fold increased risk of association with human immunodeficiency virus (HIV) infection in women living in endemic areas of SSA (Kjetland et al., 2006; Kjetland et al., 2012). There is a similar plausibility of additional risk of HIV transmission among dually-infected males in schistosomiasis-endemic areas due to observed increase in inflammatory cells and immunological mediators in semen of people with MGS which might increase the viral copies (Leutscher et al., 2005). Hence treatment of MGS with PZQ could support the control of HIV/AIDS in overlapping prevalent areas of both diseases, especially in SSA.

This systematic review on MGS in endemic areas, has the following specific objectives:

1. update the epidemiology of MGS in endemic areas,
2. review the clinicopathological features of MGS including co-infections with other diseases,
3. assess the available diagnostic techniques and treatment of MGS, and
4. determine the existing gaps to develop future research agenda of MGS.

## 2 Methods of literature review

An online literature search was conducted systematically from January 2017 to April 2018 for publications made from 1900 up to 2017, using the main search term ‘male genital schistosomiasis’ in PUBMED, EBSCOhost (CINAHL Complete, MEDLINE Complete, Global Health, eBook Collection), COCHRANE LIBRARY and WEB OF KNOWLEDGE databases, following the stipulated guidelines of each database. The main search term was combined with terms for known symptoms of MGS retrieved from the main textbooks on Tropical Medicine (Mabey et al., 2013; Farrar et al., 2014; Squire and Stothard, 2014), which included ‘haemospermia’, ‘haematospermia’, ‘ejaculate’, ‘erectile dysfunction’, ‘infertile’, ‘sterile’, ‘painful’, ‘discomfort’, ‘spermaturia’, ‘semen’. In addition, the main term was combined with terms of male genital organs, listed as ‘prostate’, ‘seminal vesicle’, ‘spermatic cord’, ‘epididymis’, ‘vas deferens’, ‘testis’ and ‘reproductive organ’.

In the PUBMED database after inputting the main search term, it automatically searched the terms as Medical subject headings (MeSH) and all fields, to produce the total results which were narrowed to those of English language. The search of the main terms in the EBSCOhost database involved all possible forms of the terms, augmented using relevant syntax ‘OR’, ‘AND’; for example, ‘male+ OR male* OR man* OR man+’ AND ‘genital+ OR genital*’ AND ‘schistosomiasis+ OR schistosomiasis* OR Schistosoma* OR *Schistosoma*+ OR bilharzia+ OR bilharzia* OR bilharziosis* OR bilharziosis+’. These terms were automatically expanded for equivalent subjects and related words, also narrowed by English language. The search in the COCHRANE LIBRARY followed a similar pattern to the EBSCOhost database. For the WEB OF KNOWLEDGE, the main terms were searched using both field tags ‘TOPIC’ and ‘TITLE’ and then combined with Booleans ‘OR’, and ‘AND’ appropriately. The results were compiled together to produce the final list of articles. Additional articles from other sources such as references from the textbooks and known parasitologists were added to the final lists from these four databases. The final articles in French and Portuguese languages were translated into English (refer to Appendix 1 Supplementary Tables).

All the articles were screened in the following five stages:

• Stage 1: Lists of articles were checked for possible duplications, which were removed.
• Stage 2: Thereafter, the titles of the remaining articles were screened for relevance to MGS, and excluded accordingly.
• Stage 3: Then, the abstracts of those remaining were read and screened for relevance to MGS. Those articles not related to MGS were removed from the list.
• Stage 4: The full-text of the remaining articles was retrieved and read through to select those manuscripts on MGS to be included in the review.
• Stage 5: The references of the full-text articles included in the review were screened for additional articles not retrieved in the above database searches.

Furthermore, leading articles on FGS and texts from prominent textbooks describing schistosomiasis (Gelfand, 1967; Jordan and Webbe, 1969; Jordan et al., 1993; Kamel and Lumley, 2004; Weiss, 2004) were read to form background knowledge and comparison to MGS where necessary. Alerts were installed on all the databases searched for this review to capture new publications and additional articles relevant to MGS.

## 3 Analysis of assembled literature

**Fig. 1:**
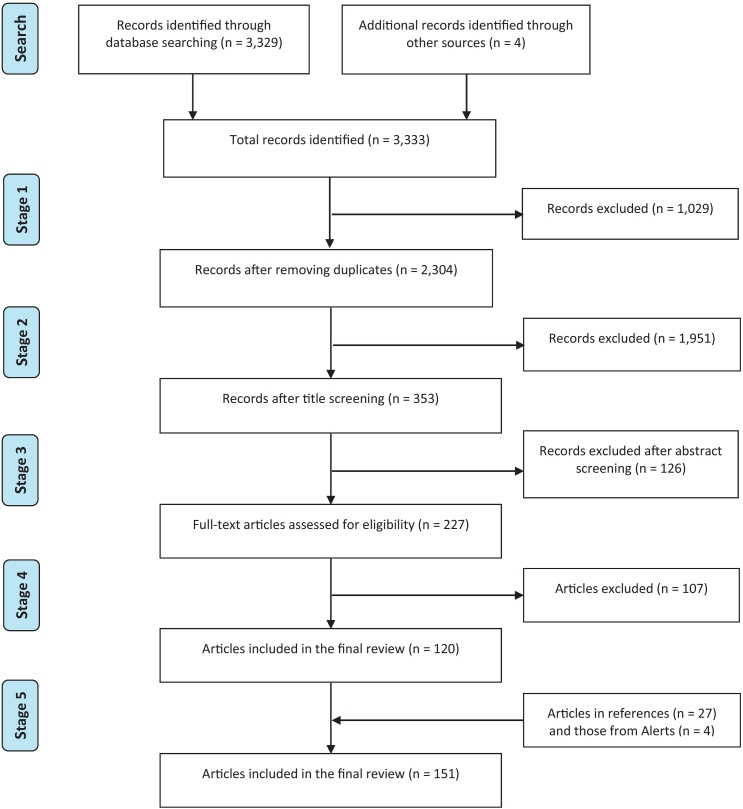
Flow chart showing the results of the systematic literature search in the online databases.

The online database search produced a combined total of 3329 publications using the main search term (Fig. 1 & Table 1). Four articles were added from the alerts on the searched databases. After screening through the five stages described earlier, the final articles included in this MGS review are 151 (Appendix 1 Supplementary Table 2), of which 32 were original research studies, 96 case reports, 2 editorial papers, 3 systematic reviews, and 18 literature reviews on schistosomiasis with aspect of male genital pathology. The period of publications was from 1911 to 2018.

**Table 1:** Results of literature search on the online databases conducted from January 2017 to April 2018.

Online database	Number of articles from each database		
	‘male genital schistosomiasis’	Combined with ‘symptoms’	Combined with ‘symptoms’ and ‘organs’
EBSCOhost	680	837	1677
PUBMED	181	339	812
WEB OF KNOWLEDGE	140	275	570
COCHRANE LIBRARY	17	96	270
TOTAL	1018	1547	3329

Thirty-two original research studies have been published from 1952, with half directly on genital schistosomiasis, while the other half focussed on schistosomiasis with brief descriptions on genital symptoms, pathologies or complications. Sixteen studies reported only on *S*. *haematobium*, 3 on *S*. *mansoni*, 9 on mixed *S*. *haematobium* and *S*. *mansoni* infections, while 4 had no mention of species (Fig. 3). In addition, 26 studies were conducted in Africa [Madagascar-6, Nigeria-6, Egypt-5, Zimbabwe-5, Zambia-2, Ghana-1] and one each in other continents except Australasia. There were 11 necropsy studies, 5 histopathological studies, 6 longitudinal cohort studies, 2 qualitative studies, 1 radiography study and 1 hormonal analysis study. Seven studies involved examination of all genital organs, 2 on seminal vesicles, 1 on prostate only while other studies did not focus on specific genital organ(s).

**Fig. 2:**
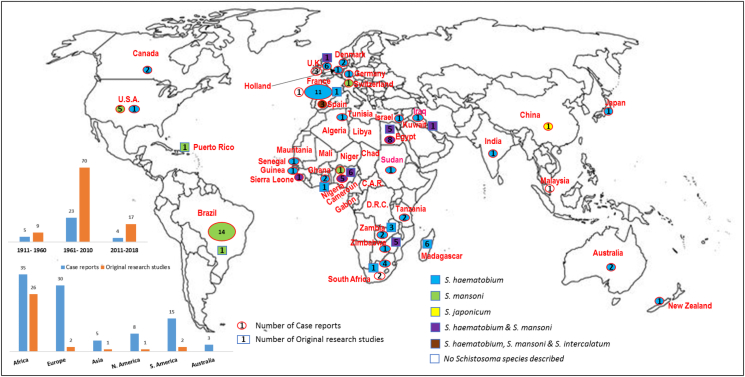
Global map showing distribution of the publications on MGS from 1911 to 2018. The two charts displaying number of publications in the first and second 50 years and per continent. (The original research studies comprise post-mortem studies conducted in Africa and South America; prospective studies mainly in Africa.)

Ninety-six case reports were made between 1911 and 2018, with only five reports published prior to 1952. Fifty-five case reports were from endemic areas mostly in Africa [n = 35; 64%] while 40 reports were on travellers or people emigrating from endemic areas to non-endemic countries, especially in Europe [n = 30; 75%] (Fig. 2). Some travellers diagnosed in non-endemic countries in Europe, Asia and Australia, were infected after swimming or walking in Lake Malawi in SSA, which is endemic mainly for *S*. *haematobium*. In France, ten of the 12 case reports were of travellers to or emigrants from North, West and Central African countries of Algeria, Cameroun, Central African Republic, Chad, Democratic Republic of Congo (DRC), Egypt, Gabon, Libya, Mali, Mauritania, Niger and Tunisia.

Sixty case reports (63%) were on *S*. *haematobium*, 22 (23%) on *S*. *mansoni*, and the rest (5%) on mixed *S*. *haematobium* and *S*. *mansoni* (n = 2), on *S*. *haematobium*, *S*. *mansoni* and *S*. *intercalatum* (n = 2), on *S*. *japonicum* (n = 1), whereas 9 (10%) had no speciation. The pathological organs described in 74 case reports included scrotum (including testis and vas deferens) [n = 39], prostate [n = 17], seminal vesicles [n = 17], spermatic cord [n = 3], epididymis [n = 7] and penis [n = 1]. The main presenting symptoms or complaints were swelling of scrotum and other genital organs [n = 58], pelvic pain [n = 23], haemospermia [n = 14], hydrocele [n = 12], changes in semen/ejaculate [n = 11], infertility [n = 6] and urethral discharge [n = 2].

The 3 systematic reviews were published in 2011 and 2015, discussing the relationship between UGS and HIV (Mbabazi et al., 2011), prostate adenocarcinoma associated with prostatic *S*. *haematobium* infection (Figueiredo et al., 2015) and MGS treatment as a future HIV prevention tool (Stecher et al., 2015).

### 3.1 Update on the epidemiology of MGS

As described earlier, *S*. *haematobium* is endemic in Africa, particularly SSA, where most knowledge originates and the first recognised description of MGS was made by Madden a century ago (Madden, 1911). However, earlier literature by Chaker, Lortet, Vialleton, Letulle and Madden [1885–1909] described lesions in genital organs like seminal vesicles and prostate which were infiltrated by schistosome ova and granulomata formation (Madden, 1909; Mensah et al., 1972; Guirassay et al., 2008). Other genital organs have been described in the subsequent reports and research studies.

Post-mortem studies were among the earliest research in endemic areas especially in Africa, describing the epidemiology of genital schistosomiasis, four decades after the Madden report, giving the background knowledge to understanding MGS (Mohammed, 1952; Gelfand and Ross, 1953; Edington et al., 1970; Gelfand et al., 1970; Edington et al., 1975). Digestive methods were performed using potassium hydroxide (KOH) to harvest the ova from the genital organs with pathologies caused by *S*. *haematobium* and *S*. *mansoni*. Seminal vesicles were infected almost as much as the urinary bladders, ranging from 50% to 80% of vesicles with over 90% of bladders (Mohammed, 1952; Gelfand and Ross, 1953) with approximately 20,000 ova found in the vesicles [Table 2] (Edington et al., 1970; Gelfand et al., 1970; Edington et al., 1975). Histopathological examinations were also conducted, in other studies to compare with the digestive methods which showed that more ova were observed with the latter technique (Gelfand et al., 1970).

**Table 2:** Total number of *Schistosoma* ova in pelvic organs in necropsy studies.

Study participants	Post-mortem studies	
	Gelfand et al., 1970	Edington et al., 1975
Total number	200	54


Pelvic organs	Intensity of *Schistosoma* egg distribution	
	Gelfand et al., 1970	Edington et al., 1975
Bladder	105,011	13,260–87,100
Seminal vesicles	19,801	4312–12,027
Vas deferens	2913	–
Prostate	34	169–9828

In endemic areas of *S*. *haematobium* and *S*. *mansoni*, the former predominates with more genital pathologies in literature than the latter, similarly to the case reports (Grace and Aidaros, 1952; Alves et al., 1955). From these studies, it has been described that MGS affects between 1% to 20% of those in endemic areas at risk and suffering from UGS (Ricosse et al., 1980; Fievet et al., 1984). This could be a gross underestimation, because several studies have reported that at least 50% of genital organs are infected by schistosome ova, emphasising that MGS is as common as urinary manifestations of schistosomiasis but with lower intensity of ova [Table 3] (Edington et al., 1970; Edington et al., 1975; Elem and Patil, 1987; Patil and Elem, 1988).

**Table 3:** *Schistosoma* ova in male genital organs seen in necropsy studies.

Year	Author(s)	Country	Autopsies	Species	Infected genital organs
1955	Alves et al.	Zimbabwe	50	*Sh*, *Sm*	18% vas deferens; 18% prostate; 4% tunica vaginalis; 2% epididymis
1956	Arban	Brazil	3233	*Sm*	10/3233 infected: 20% prostate; 30% testes
1970	Gelfand et al.	Zimbabwe	200	*Sh*, *Sm*	54% seminal vesicles; 39.9% spermatic duct; 20.5% prostate
1975	Edington et al.	Nigeria	54	*Sh*	Severe infections: 100% prostate; 100% seminal vesicles; 57% testes; 57% epididymis
1987	Elem & Patil	Zambia	50	*Sh*	62% bladder; 58% seminal vesicles; 50% prostate
1988	Patil & Elem	Zambia	100	*Sh*	62% bladder; 58% seminal vesicles; 50% prostate

*Sh - S*. *haematobium*; *Sm - S*. *mansoni*.

The first identified prospective study on MGS was conducted in Madagascar in 1999–2000, where 19 of 44 participants (43%) had MGS by *S*. *haematobium* ova in semen (Leutscher et al., 2000). Although the sample size of this study was small, subsequent longitudinal studies in the same country showed similar prevalence of MGS, ranging from 28% in 2005 to 53% in 2009 (Leutscher et al., 2005; Leutscher et al., 2008a; Leutscher et al., 2008b; Leutscher et al., 2009). *Schistosoma* ova were present in semen only in some cases, highlighting fact that the prevalence of MGS is quite significant, similarly to that of UGS in endemic areas, despite not having been studied as extensively.

### 3.2 Clinico-pathological features of MGS including co-infections

From our search, genital organs with schistosomal pathologies have been recorded in case reports from Africa, namely prostate, seminal vesicles, vas deferens, testis and scrotum which were more associated with *S*. *haematobium* than *S*. *mansoni* (Cerqua, 1930; Mohammed, 1930; Makar, 1937; Gelfand and Davis, 1940). An early report from South America associated with *S*. *mansoni*, presented of enlarged scrotum, thickened seminal vesicles and hydrocele (Armbrust, 1951). Subsequent reports indicate that a higher burden of MGS is in *S*. *haematobium* - endemic areas of Africa than other schistosome - endemic areas in the world.

Although most of the MGS pathologies have been reported on *S*. *haematobium* in inhabitants and travellers to endemic areas, similar reports have been made on *S*. *mansoni*, *S*. *intercalatum* and *S*. *japonicum* (Corachan et al., 1994; Vilana et al., 1997; Yu et al., 2013). Infestation of genital organs results in several early symptoms of MGS. One major symptom observed in early stages is haemospermia resulting from egg penetration and release into seminal vesicle lumen, causing ulceration of mucosal lining, and pain during coitus and ejaculation (Madden, 1911; Makar, 1937; Mohammed, 1952; Kato-Hayashi et al., 2013; Lang et al., 2017). Haemospermia can occur as the only symptom or first symptom preceding haematuria, occurring within three months of exposure to infection (Becquet, 1966; Pedro Rde et al., 1973; Corachan et al., 1994; Schwartz et al., 2002).

Of interest, this symptom has been described more frequently among travellers than inhabitants of endemic areas, in 8 of the 12 case reports found in the search. This could be due to failure to recognise the symptoms, societal acceptance of condition as male menstruation and maturing from boyhood to adulthood, not knowing or making an association with MGS, being mistaken with sexually transmitted infections (STIs) or infidelity (Ukwandu and Nmorsi, 2004; Yirenya-Tawiah et al., 2016). In relation to haemospermia, other reported symptoms include alteration in semen quality and appearance with discolouration (McKenna et al., 1997; Torresi et al., 1997; Hawary et al., 2012), subjective change (Davies and Hamdy, 1998), lumpy semen (Lewis et al., 1996; Lang et al., 2017), rice grains with increased volume (Pedro Rde et al., 1973) and reduced viscosity or volume (Perignon et al., 2007; van Delft et al., 2007; Knapper et al., 2012).

The symptoms associated with mucosal thickening and enlargement of organs such as seminal vesicles cause irritation of the sympathetic nervous system leading to sexual hyperexcitability, night dreams and frequent painful erections (Mohammed, 1952). However, these symptoms have not been reported in the last four decades, raising the question of their reliability in the earlier studies or non-reporting in the recent studies, possibly due to the sensitive descriptive nature. The enlargement of genital organs has also been mistaken for other diseases such as tuberculosis or malignancy resulting in extraneous surgical interventions where PZQ treatment provided earlier might have prevented the surgery (Madden, 1911; Chippaux et al., 1957; Eltayeb, 1969; Kazzaz and Salmo, 1974; Fievet et al., 1984; Githae, 1992; Ferreira et al., 2015). Untreated, the organs chronically become nodular, firmer, smaller and non-functional.

More recently reported symptoms of MGS include spermaturia (sperm in urine) as a result of fibrosis and abnormal cystic dilatation of seminal vesicles (Etribi et al., 1967), hydrocele formation (Gelfand and Davis, 1940; Armbrust, 1951; Van Beukering and Vervoorn, 1956; Pawel et al., 2008; Ramarakoto et al., 2008; Rambau et al., 2011), epididymitis (Alves et al., 2004), funiculitis (Durand et al., 2004), orchitis (Mikhail et al., 1988; Ihekwaba, 1992; Al-Qahtani and Droupy, 2010), prostatitis (Cerqua, 1930; Alexis and Domingo, 1986; Patil and Elem, 1988; Cohen et al., 1995; Fender et al., 1996; Basilio-de-Oliveira et al., 2002; Al-Saeed et al., 2003; Lambertucci et al., 2006; Bacelar et al., 2007; Sharma et al., 2015), infertility from oligospermia, azoospermia either obstructive from blockage of vas deferens, spermatic cord, epididymis, tunica vaginalis or non – obstructive through infarction (Kini et al., 2009; Abdel-Naser et al., 2018a; Abdel-Naser et al., 2018b), fibrotic lesions (Edington et al., 1975) or functional lymphotic infiltration in testis (Adisa et al., 2012). While egg load in the bladder tissue has been found to correlate with pathological severity, few ova in seminal vesicles, prostate and other genital organs have been associated with severe extensive pathological changes (Edington et al., 1970; Edington et al., 1975).

On malignancies of genital organs, our search showed that MGS has been reported among travellers and those emigrating from endemic areas, apart from testicular or scrotal schistosomiasis simulating neoplasia (Alexis and Domingo, 1986; Cohen et al., 1995; Ma and Srigley, 1995; Basilio-de-Oliveira et al., 2002; Bacelar et al., 2007; Lopes et al., 2007; Figueiredo et al., 2015). Prostatic adenocarcinoma has been observed to occur together with *Schistosoma* ova, resulting in epithelial granulomata, marked fibrosis and organ enlargement, which have been described in reports of tissue histopathology and cancer spread to other genital organs affected by MGS. Despite an accepted link between chronic UGS and squamous cell carcinoma of the bladder (Honeycutt et al., 2014), the mechanism of association between prostatic cancer and schistosomiasis remains unknown.

**Fig. 3:**
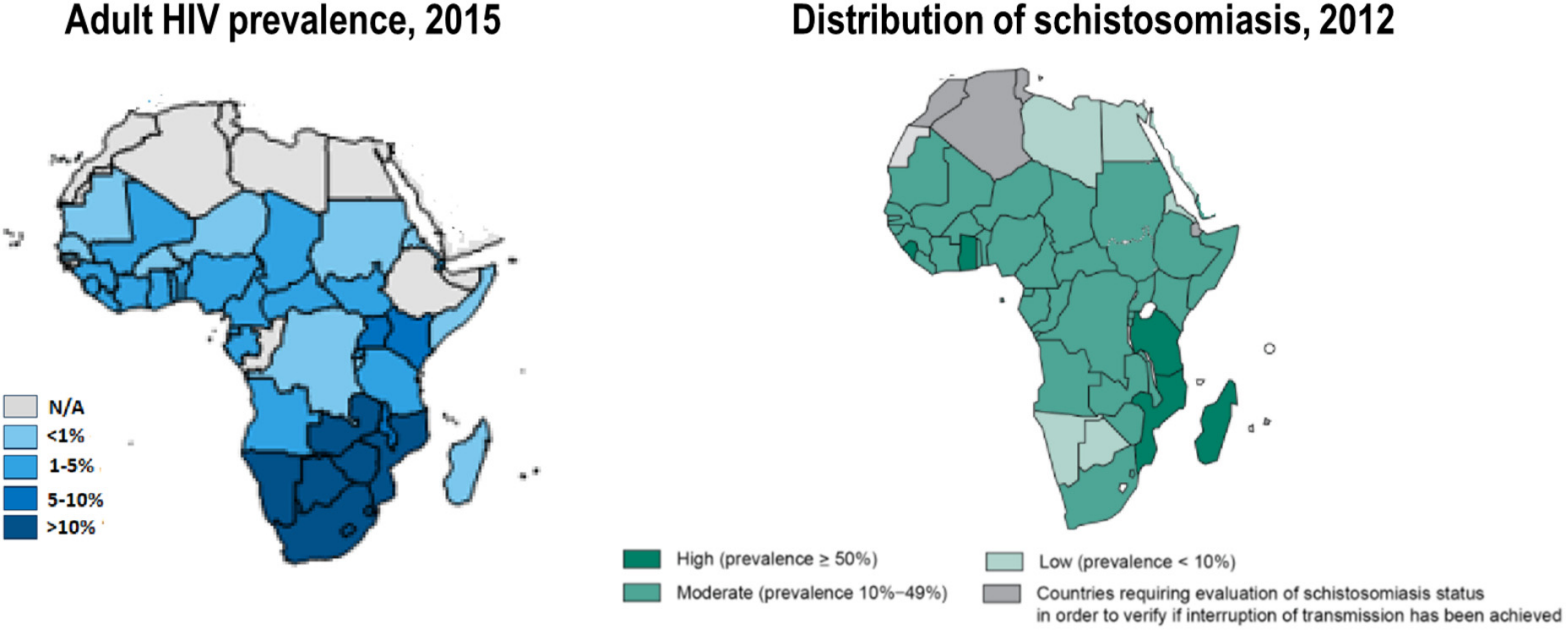
Map of Africa showing the correlation of the prevalence of HIV and schistosomiasis. Produced from (WHO, 2014; Kaiser-Family-Foundation, 2016).

Our search produced recent systematic reviews addressing extensively the interactions of both MGS and HIV and were included in this review (Mbabazi et al., 2011; Stecher et al., 2015). As one of the leading causes of morbidity and mortality in the world, HIV has its epicentre in the SSA region (UNAIDS, 2016) where coincidentally schistosomiasis is endemic (Fig. 3). Female genital schistosomiasis (FGS) has been observed in 33–75% of women having UGS living in endemic areas in SSA (Kjetland et al., 2012). In addition, FGS has been associated with a 3-fold increased risk of HIV infection with characteristic sandy-grainy patches present in egg-infected genital organs, abnormal blood vessel formation and increased levels of inflammatory cells expressing CD4+ receptors triggered by *Schistosoma* granulomata (Kjetland et al., 2006; Christinet et al., 2016). More research and gleamed knowledge on the risk and interplay of MGS with HIV infection is needed.

Various hypotheses have been proposed regarding the impact of MGS on HIV transmission. As described in Leutscher et al. (2005) study and the systematic reviews by Mbabazi et al. (2011) and Stecher et al. (2015), men with MGS have elevated levels of eosinophils and lymphocytes among other inflammatory cells expressing CD4+ receptors together with cytokines IL-4, 6, 10, TNF-α. These recruit more HIV-infected cells into semen, upregulating viral replication and increasing viral concentration (Leutscher et al., 2005; Mbabazi et al., 2011; Stecher et al., 2015). With the chronic inflammation and cell recruitment to the male genital tract, these may increase HIV viral load in semen, similar to that seen with STIs (Mabey, 2000). A recent observational pilot study in endemic SSA area demonstrated a reduction of viral load shedding in semen of HIV positive men coinfected with UGS 10 weeks after PZQ treatment (Midzi et al., 2017). Further case-cohort or randomized studies are needed to be conducted in endemic areas to explore these critical findings further.

### 3.3 Techniques for detection of MGS

Urine microscopy remains the definitive way for identifying schistosome ova (mainly of *S*. *haematobium*) to diagnose UGS. While it is the gold standard (Le and Hsieh, 2017) and also considered as a useful proxy for diagnosing MGS, our findings indicate challenges in its reliability due to presence of ova in semen or histological specimens in the absence of ova in urine (or stool), as well as other schistosome species that may on occasion cause MGS (Corachan et al., 1994; Torresi et al., 1997; Leutscher et al., 2000; Schwartz et al., 2002; Lang et al., 2017). As such, there is a direct need to conduct microscopy on semen and biopsy material from suspicious genital lesions/tissues to diagnose MGS. Also, semen should be analysed repeatedly with periods of abstinence to cater for the daily diurnal variations in excretion of ova from the genital organs and increase likelihood of maximum egg yield, similar to the recommended consecutive urine analyses (Leutscher et al., 2008b; Le and Hsieh, 2017). However, local perceptions, beliefs and sensitivity of people in endemic communities needs careful consideration with regards to ejaculation and handling semen samples. Combined this can affect the collection or submission and later analyses, thus to optimise the process there is usually a need for more health education and counselling of those required to submit as well as within the community to ensure acceptability and success in engagement alongside collection of the samples in the community (Price et al., 2005; Midzi et al., 2017).

Leutscher and colleagues report on use of eosinophil cationic protein (ECP), circulating anodic antigen (CAA) and soluble egg antigen (SEA) as blood-based markers of MGS which showed positive correlation to urine egg count, with ECP significantly correlating with urine count and declining after PZQ treatment, highlighting its importance in diagnosis (Leutscher et al., 2000; Leutscher et al., 2008b). However, other helminth, bacterial or viral infections and inflammatory conditions elevate ECP hence affecting the reliability in co-morbidities which are common in endemic areas. PCR and DNA-based tests on the other hand have shown to be highly sensitive and specific diagnostic tools, which can be used in urine, semen, and many other specimens (Le and Hsieh, 2017). These tests are still expensive for field use in endemic areas of Sub-Saharan Africa, hence the need to develop easier, accessible, low-cost tests.

Our findings showed that ultrasonography is useful in diagnosing lesions caused by MGS and monitoring morbidity of the pathologies (Richter, 2000; Al-Saeed et al., 2003; Ramarakoto et al., 2008). The pathological lesions seen in genital organs have been described as echogenic lesions and calcifications, with the former improving with treatment (Richter, 2000; Ramarakoto et al., 2008). Availability of portable sonography machines could be more cost-effective in endemic areas since other radiological techniques such as computerised tomography (CT) and magnetic resonance imaging (MRI) are very expensive, not feasible and almost non-existent in these regions. However, there has been limited radiological research on MGS in endemic areas, hence there is a need for more field studies to study the resolution of pathologies after treatment.

### 3.4 Current treatment options for MGS

Praziquantel (typically offered at 40 mg/kg) has remained the mainstay treatment for most forms of schistosomiasis, including MGS (WHO, 2013). It is effective with population cure rates of over 90% and targets adult worms thereby reducing egg excretion and averting morbidity, however, praziquantel does not successfully kill juvenile worms (Rollinson, 2009). Most identified case reports and studies used the recommended traditional dosage of 40 mg/kg in treating MGS with some failure cases requiring further repeated doses or higher dose of 60 mg/kg (Schwartz et al., 2002; Alonso et al., 2006; Perignon et al., 2007). It has been suggested that higher doses are more efficacious in MGS treatment than the traditional dose alongside shorter intervals between retreatments, for example, 2–3 times a year (Lang et al., 2017).

Use of PZQ in most African programmes is based on morbidity control through mass drug administration versus specific case management. The former is an attempt to keep prevalence and intensity down to an acceptable level, below 10% in the endemic population and obtain the greatest cost-benefit outcome at population level. There are well-known gaps in this approach, for example, school-aged children are targeted with donated stocks of PZQ ring-fenced (restricted) for this use in school-based programmes. As an indirect consequence, many adolescents and adults rarely receive adequate treatment and PZQ is not always available in peripheral health clinics which further affects management of schistosomiasis in an individual case management setting (Christinet et al., 2016; McManus et al., 2018).

### 3.5 Existing gaps for further research of MGS

This review conducted a systematic search to elucidate the burden of MGS in endemic areas, a century after the first recognised report in 1911. Despite the detailed epidemiology of schistosomiasis in the world highlighting the enormous impact of UGS, much remains unknown of the burden of genital manifestations of schistosomiasis either FGS or MGS, specifically. More description and research studies of UGS especially in endemic areas have concentrated on urinary system and associated pathologies, however, with the growing interest in cervical cancer screening there are opportunities to integrate surveillance of FGS (Christinet et al., 2016). On the other hand, for men, no such screening programmes exist and therefore the prevalence and morbidity of MGS in endemic areas will remain under-reported.

In addition, the limited description of MGS is compounded by difficulties in diagnostic techniques and approaches, these include deficits in standardised protocols for analysis of semen. Indeed, future methods which involve molecular assays will be challenging to carry out in primary health facilities in SSA. Future research studies to explore the deployment of low-cost techniques and methods are urgently required. These would be particularly important regarding treatment and management of MGS, as currently there is a clear gap in our understanding of the optimal dose of PZQ to treat MGS, whether single, repeated (i.e. 2–3 times a year) or higher dosages (i.e. >60 mg/kg) would be effect a parasitological cure (Schwartz et al., 2002; Alonso et al., 2006; Lang et al., 2017), notwithstanding tracking the dynamics of lesions in the genital tract. This highlights the need for further prospective longitudinal studies in endemic areas and more clinical research exploring an agenda of how best to integrate preventive treatment and management of MGS alongside ongoing interventions for HIV in SSA.

## 4 Discussion

This review has revealed that genital organs are infested with schistosome ova in the early stages of the infection, similar to other forms of the disease. These organs are infected with substantial numbers of ova as much as urinary bladder or intestines, further indicating the higher levels of MGS in endemic areas. Clinical manifestations associated with MGS in this review have been described previously by Barlow after self-infection with cercariae (Barlow and Meleney, 1949) and are regarded as major symptoms and diagnostic for MGS. Symptoms like haemospermia can also present in other diseases such as hypertension, prostatitis or STIs (Feldmeier et al., 1999), raising the need to exclude other conditions before concluding the diagnosis of MGS. Underreporting and misconceptions of these symptoms which may have negative perception in the community, contribute to misdiagnosis and underestimation of MGS in these endemic areas (Ukwandu and Nmorsi, 2004; Yirenya-Tawiah et al., 2016). Furthermore, co-existence of MGS and prostatic metaplasia and malignancies require further research to understand the link, and develop diagnostic and therapeutic interventions.

Although urine microscopy has been considered as a proxy for diagnosing MGS, our findings observed challenges of ova found only in semen without any in urine or stool, hence the need to consider semen microscopy as a definitive way of diagnosing MGS. Accessible, low-cost molecular tests should be developed to address this diagnostic challenge. Similarly, radiological techniques like field-based ultrasonography should be rolled out into endemic areas to monitor the morbidity and resolution of MGS pathologies. MGS appears to be prevalent in areas endemic for UGS, which coincidentally are high prevalent areas for HIV. Some people in these areas in SSA have higher HIV prevalence and also at higher risk for schistosomiasis due to their lifestyles and daily activities, as reported about fishermen in Malawi (NSO, 2014; NAC, 2015). With some evidence of MGS potentially upregulating viral replication, increasing the concentration of HIV particles in the semen and exponentiating the infectiousness of dually infected males, treatment of MGS could be an importance tool in helping to avert new HIV infections in SSA (Stecher et al., 2015). Interestingly, one of the current effective intervention of HIV prevention, male circumcision, was considered by ancient Egyptians around 2300 BCE as an intervention to prevent schistosomal infection among men bathing in infested waters, though later disputed (Allen, 1909; Madden, 1919; Jordan, 2000; Weiss, 2004).

## 5 Conclusion

MGS is an under-appreciated manifestation of UGS and has been reported worldwide but its current distribution is most tightly linked with areas endemic for *S*. *haematobium*. In SSA, MGS likely blights the lives of millions of men who currently do not have adequate access to point-of-care diagnostics or access to optimal praziquantel treatment regimes. We propose that MGS should be considered specifically in a new light of individual case management approaches as being used for other NTDs.

## Appendix A Supplementary data

Supplementary tables Image 1
