# Supplementary material for: A systematic review with epidemiological update of male genital schistosomiasis (MGS): A call for integrated case management across the health system in sub-Saharan Africa
Source: Parasite Epidemiol Control. 2018 Nov 23;4:e00077. doi: 10.1016/j.parepi.2018.e00077 (PMC6324017; doi:10.1016/j.parepi.2018.e00077)
Supplement: Supplementary file 1 — Supplementary tables [file mmc1.doc]

# Appendix 1: Supplementary Tables

## Table 1: Additional articles on MGS received from Parasitologists and Alerts from databases

| **Articles added to those retrieved from online search before the screening stages** | | | |
| --- | --- | --- | --- |
| **No.** | **Authors** | **Year** | **Key facts** |
| 1. | Barlow and Meleney | 1949 | Voluntary schistosomal infection with haemospermia in early stages |
| 2. | Feldmeier *et al.* | 1999 | Male genital schistosomiasis and haemospermia |
| 3. | Rollinson | 2009 | Review of history, life-cycle and clinic-pathologies of schistosomiasis |
| 4. | Le and Hsieh | 2017 | Review of current diagnostics, challenges and future advances |
|  | | | |
| **Articles added from the Alerts on the online searched databases after the screening stages** | | | |
| 1. | Midzi *et al.* | 2017 | Decreased in seminal HIV-1 viral load after PZQ treatment of UGS coinfection in HIV-positive men |
| 2. | Aber-Naser *et al.* | 2018 | Testicular schistosomiasis in an obstructive azoospermic man |
| 3. | Mcmanus *et al.* | 2018 | Review of the epidemiology, pathophysiology, diagnosis, management and control of schistosomiasis, some facts on genital manifestations |
| 4. | Aber-Naser *et al.* | 2018 | Review of schistosomiasis as an important cause of male infertility in endemic areas |

## Table 2: All articles included in the review

| **Original research studies** (* those focussed specifically on MGS; ^ǂ^ those from references) | | | | | |
| --- | --- | --- | --- | --- | --- |
| **No.** | **Authors** | **Year** | **Country** | **Organs / Specimens** | **Key findings** |
| 1. | Mohammed* | 1952 | Egypt | Post-mortems:  Seminal vesicles  Prostate | 80% seminal vesicles and 19% prostates infected with bladder; dense fibrosis and calcified eggs |
| 2. | Grace and Aidaros*^ǂ^ | 1952 | Egypt | Autopsies:  Seminal vesicles | 95% seminal vesicles involved in infected autopsies, bilateral, 94% with *S. haematobium* calcified eggs |
| 3. | Gelfand and Ross^ǂ^ | 1953 | Zimbabwe | Post-mortems:  all organs, males and females | *S. haematobium* more common than *S. mansoni* n prostate despite rectum close proximity |
| 4. | Alves *et al.** | 1955 | Zimbabwe | Autopsies: male genital organs | 76% of autopsies showed bilharzial eggs; prostate, vas deferens, seminal vesicles involved most. |
| 5. | ArbÁN | 1956 | Puerto Rico | Autopsies and surgical removed specimens | 10 genitourinary lesions due to *S. mansoni*; kidneys, bladder, testes, prostate and seminal vesicles |
| 6. | King | 1965 | South Africa | Urine microscopy  Radiological exam | More epididymo-orchitis in no urine eggs than with; prostatitis in 0.11% with eggs, 0.16% without |
| 7. | Etribi *et al.** | 1967 | Egypt | Histopathological analyses of thick vas deferens | 62.7% of subfertile men had Bilharziasis (eggs in semen, urine, calcifications), obstructive testicles |
| 8. | Gelfand *et al.** | 1970 | Zimbabwe  (S. Rhodesia) | Autopsies: prostate, seminal vesicles, vas deferens | Seminal vesicles, spermatic cord, prostate infected more with *S. haematobium* than *S. mansoni* eggs |
| 9. | Edington *et al.* | 1970 | Nigeria | Autopsies: all organs | Mean egg load more in males; eggs in seminal vesicles, prostate, vas deferens, epididymis; calcified. |
| 10. | Edington *et al.** | 1975 | Nigeria | Autopsies; male and female genital organs, appendix, brain | Seminal vesicles, prostate, testes, epididymis were most infected; atrophy, fibrosis with calcified eggs |
| 11. | Aboul *et al.** | 1977 | Egypt | Seminal vesicles | Seminal vesiculitis due to severe bilharzial infiltration showed no obstructive infertility. |
| 12. | Cheever *et al.* ^ǂ^ | 1978 | Egypt | Autopsies: pathology of extrahepatic organs | Active lesions in seminal vesicles, prostate and ejaculatory ducts; eggs, fibrosis, muscle hypertrophy |
| 13. | Ricosse *et al.* | 1980 | France | Pathological specimens | Testes: hard, irregular, hydrocele  Epididymis: nodular mass, inflamed |
| 14. | Bello and Idiong | 1982 | Nigeria | - | 42.2% of the urethral discharge students had *S. haematobium* eggs |
| 15. | Gwavava *et al.* ^ǂ^ | 1984 | Zimbabwe | Surgical biopsies from all organs | Penile ulcer, swollen testis / epididymis, hydrocele hematuria |
| 16. | Elem and Patil* | 1987 | Zambia | Autopsies: bladder, seminal vesicles, prostate; urine, semen | 11 hemospermia men - no eggs; 100 semen – no eggs but 2 in urine; 58% prostate, 50% seminal vesicles |
| 17. | Patil and Elem* | 1988 | Zambia | Post-mortems: bladder, seminal vesicles, prostate | Digestion vs histology exams: s. vesicles – 50% vs 25%, prostate- 50% vs 9%, bladder – 62% vs 20% |
| 18. | Ogunbanjo *et al.* | 1989 | Nigeria | - | 2/54 seminal fluids from infertile men had *S. haematobium* eggs |
| 19. | Skelly *et al.** | 1994 | Brazil | - | No significant differences in testosterone levels between infected and non-infected males |
| 20. | Leutscher *et al.** | 2000 | Madagascar | - | 57% with urine eggs, 43% in semen ECP in urine and semen correlated with number of eggs excreted |
| 21. | Al-Saeed *et al.* | 2003 | Kuwait | Seminal vesicles | Solid masses of seminal vesicles observed on prostate transrectal sonography; chronic infection |
| 22. | Ukwandu and Nmorsi | 2004 | Nigeria | - | 66% with eggs in urine; 4.3% in semen; 22% had sexual pain. |
| 23. | Leutscher *et al.** | 2005 | Madagascar | - | 67% in urine, 28% semen; cytokines, leucocytes, lymphocytes increased; declined with PZQ |
| 24. | Mohammed *et al.* | 2007 | Nigeria | Endoscopic or surgical biopsies (GIT, GUT) | 10% male genital organs affected: prostate, testis, epididymis |
| 25. | Leutscher *et al.** | 2008 | Madagascar | - | 62% eggs in urine, 42% in semen; ECP, SEA, CAA correlated with egg count, with CAA performing well |
| 26. | Leutscher *et al.* | 2008 | Madagascar | - | 55% eggs in urine; 17% had STI; urethral discharge, haematuria, dysuria, painful ejaculate. |
| 27. | Ramarakoto *et al.** | 2008 | Madagascar | Urogenital organs with *S. hematobium* | Hyperechogenic and calcified lesions in seminal vesicles and prostate; enlarged |
| 28. | Leutscher *et al.** | 2009 | Madagascar | - | 53% egg in semen, lower volume, leukocytospermia; apoptosis correlate with ECP, lower with PZQ |
| 29. | Coltart *et al.* | 2015 | UK | - | 6/10 patients with haematospermia had eggs in urine |
| 30. | Yirenya-Tawiah *et al.* | 2016 | Ghana | - | 94.4% males knew UGS, sexual dysfunction, urethral discharge, haemospermia, itchy scrotum; 12.3% UGS help HIV acquisition |
| 31. | Lima *et al.* | 2017 | Brazil | Histopathological diagnosis for ectopic forms of *S. mansoni* | 5 cases of male genital *S. mansoni*: testis (3, 2 had orchiectomy for prostatic adenocarcinoma; 1 with chronic orchitis); penis (1) with squamous cell carcinoma; epididymis (1) with epididymitis. |
| 32. | Midzi *et al.* | 2017 | Zimbabwe | Urine & stool for schistosomiasis diagnosis; blood for HIV-1 diagnosis & RNA viral load monitoring; semen for RNA viral load monitoring | 18 HIV-positive men (6 ART-naïve & 12 ART-experienced) with *S. haematobium* in urine recruited and treated at baseline; followed up for 10 weeks. Mean HIV viral load reduction on follow-up after PZQ & no *S. haematobium* in urine |

| **Case reports** (^#^Letters to the Journal Editors, ^ǂ^ those from references) | | | | | |
| --- | --- | --- | --- | --- | --- |
| **No.** | **Authors** | **Year** | **Country** | **Organs / Specimens** | **Key findings** |
| 1. | Madden^ǂ^ | 1911 | Egypt | Right scrotum Spermatic cord  Epididymis  Seminal vesicles | Hard, painless swelling; epididymis replaced by nodules full of eggs.  Haemospermia, hematuria, pain, thick seminal vesicle, eggs in urine |
| 2. | Cerqua^ǂ^ | 1930 | Egypt | Prostate | Frequency, scalding erection; painless elastic swelling with eggs |
| 3. | Mohammed^ǂ^ | 1930 | Egypt | Seminal vesicle  Vasa deferentia | *S. haematobium* in urine; both organs fused with calcified eggs |
| 4. | Makar^ǂ^ | 1937 | Egypt | Prostate  Seminal vesicles  Urethra | Haemospermia; enlarged prostate, seminal vesicles, calcifications, *S. haematobium* eggs in urine, semen |
| 5. | Gelfand and Davis^ǂ^ | 1940 | Zimbabwe | Left testis  Epididymis | Enlarged, knobby testis; hydrocele, obliterated epididymis; also, eggs |
| 6. | Armbrust^ǂ^ | 1951 | Brazil | Scrotum | Enlarged scrotum, hydrocele with dark fluid, *S. mansoni* in testes |
| 7. | Van Beukering and Vervoorn | 1956 | Ghana  (Gold Coast) | Scrotum | Infertility; thick funiculi; bilateral hydrocele with eggs; azoospermia |
| 8. | Chippaux *et al.* | 1957 | France | Seminal vesicles | Haematuria, mass near seminal vesicle, with *S. haematobium* eggs |
| 9. | Piganiol *et al.* | 1957 | Senegal | Seminal vesicles | 2-3 times increase in volume and hypertrophy of seminal vesicles |
| 10. | Joshi^ǂ^ | 1962 | Ghana | Scrotum | Bilateral painless swelling; mass with testis, epididymis and cord fused, infarcted with calcified eggs |
| 11. | Cayret *et al.* | 1963 | France | Seminal vesicles | 7 cases presenting with hematuria, pelvic pain, enlarged vesicles, orchitis |
| 12. | Klerk | 1964 | South Africa | Seminal vesicles | Dysuria, groin pain, firm seminal vesicles felt; calcified, thickened |
| 13. | Houston | 1964 | Zimbabwe  (S. Rhodesia) | Right testis | Enlarged abnormal; with bladder; having bilharzial pseudo-tubercles |
| 14. | Chaves and Figueiredo | 1965 | Brazil | Scrotum | Pruritic eruptions, ulcer and edema; nodules, papules with eggs |
| 15. | Becquet | 1966 | France | - | Hemospermia, viable *S. haematobium* eggs in seminal fluid |
| 16. | Joshi | 1967 | Sierra Leone | Right scrotum | Painless hard swelling; calcified eggs and fibrosis |
| 17. | Eltayeb | 1969 | Sudan | Right testis Epididymis | Painless swelling, non-tender irregular with hard nodules and hydrocele; many eggs in epididymis |
| 18. | Chatelain *et al.* | 1969 | France | Epididymis | Haematuria, dysuria, urethral shrinkage; fistulae lesion left epididymis, large seminal vesicle |
| 19. | Rosenberg *et al.* | 1971 | France | Left testicle | Painless testicular mass with dilated calcified urethra, urine eggs |
| 20. | Mensah *et al.* | 1972 | Senegal | Prostate  Epididymis | Perineal pain, hematuria, enlarged prostate, hydrocele, discharge |
| 21. | Richaud *et al.* | 1972 | France | Right scrotum | Nodule of cord lower part with eggs and sclero-inflammation |
| 22. | Monnet *et al.* | 1972 | France | Left testicle | Swelling of left testicle in a 12-year-old boy from Tunisia |
| 23. | Pedro Rde *et al.* | 1973 | Brazil | - | Haemospermia, watery red-brown semen with rice grains, many eggs |
| 24. | Kazzaz and Salmo^ǂ^ | 1974 | Iraq | Epididymis | Enlarged nodular swelling, multiple granuloma, calcified eggs, fibrosis |
| 25. | Nwokolo | 1974 | Zambia | - | Purulent urethral discharge |
| 26. | Steinberger *et al.* | 1975 | U.S.A. | Left scrotum | Left groin pain and swelling; necrosis and granuloma with eggs |
| 27. | Mampilly and Sunkwa-Mills ^ǂ^ | 1976 | Zambia | Scrotum | Left inguino-scrotal swelling; thickened epididymis and spermatic cord |
| 28. | Badejo *et al.* ^ǂ^ | 1978 | Nigeria | Penis | Ulceration of penis with eggs in dermis; almost autoamputation |
| 29. | Elbadawi *et al.* ^ǂ^ | 1978 | U.S.A. | Left testicle | Firm enlarged testis and prostate; tubular hyaline with calcified eggs |
| 30. | Adeyemi-Doro *et al.* | 1979 | Nigeria | Perineum | Numerous, discrete, firm, painless papular eruptions; biopsy with viable and calcified *S. haematobium* eggs |
| 31. | Lembeli and Venkataramaiah^#^ | 1981 | Tanzania | - | *S. haematobium* eggs in semen |
| 32. | Peyer and Graber | 1982 | Switzerland | Testis | Testicular pain, hematuria; budding lesions with eggs and inflammation |
| 33. | Fievet *et al.* ^ǂ^ | 1984 | France | Right testicle | nodule inferior pole; hypoechoeic, epididymal nodule with pus, eggs |
| 34. | Alexis and Domingo | 1986 | U.S.A. | Prostate | Urinary obstruction; *S. mansoni* eggs with adenocarcinoma |
| 35. | Bambirra *et al.* | 1986 | Brazil | Right testicle | Enlarged firm testis, nodule, granulomas, viable and dead eggs |
| 36. | Bac *et al.* | 1987 | South Africa | Left scrotum | Haematuria, hydrocele, firm irregular enlarged testis, nodules with many viable and calcified eggs |
| 37. | Hamida *et al.* | 1987 | Tunisia | Seminal vesicles | Back pain, dysuria, hematuria, hypogastric mass; no urine eggs; cyst in right vesicle and eggs in muscularis |
| 38. | Mikhail *et al.* | 1988 | Egypt | Right scrotum  Seminal vesicles  Spermatic cord | Swelling, heaviness, sensation loss, hydrocele; *S. haematobium* and *S. mansoni* eggs in testis |
| 39. | Elem *et al.* | 1989 | Zambia | Right testis  Epididymis | Painless enlarged testis; nodules on fibrocellular mass with eggs |
| 40. | Fataar *et al.* | 1990 | New Zealand | Seminal vesicles | Seminal vesicle calcifications and granuloma seen on CT scan |
| 41. | Wedel and Jess | 1991 | Denmark | Right testis | Haematuria, granulomas with eosinophils and necrosis plus eggs |
| 42. | Ihekwaba | 1992 | Nigeria | Left testes | Enlarged left testes; hydrocele; granuloma with calcified eggs |
| 43. | Githae | 1992 | South Africa | Left testicle | Left hydrocele, hard, irregular non-tender testis, granuloma formation |
| 44. | Bornman *et al.* | 1992 | South Africa | - | Primary infertility; *S. haematobium* eggs in semen, low sperm motility |
| 45. | Fall *et al.* | 1992 | Senegal | Left testicle | Pelvic pain, hematuria; painless, firm, multinodular testis; orchidectomy with *S. haematobium* eggs found. |
| 46. | Godec *et al.* ^ǂ^ | 1992 | U.S.A. | Prostate | Lower back pain, diffuse indurated prostate; bone metastases and prostate adenocarcinoma with *S. mansoni* eggs |
| 47. | Obel and Black | 1994 | Denmark | - | Left inguinal pain; thin clotting semen with numerous *S. haematobium* eggs, none found in urine |
| 48. | Corachan *et al.* | 1994 | Spain | Prostate  Seminal vesicle | Hematospermia, perineal and coital discomfort, calcifications in prostate and seminal vesicle |
| 49. | Cohen *et al.* | 1995 | South Africa | Prostate | Elevated PSA, adenocarcinoma, with *S. haematobium* viable and calcified eggs; also adult worms |
| 50. | Ma and Srigley | 1995 | Canada | Prostate  Seminal vesicles | Both enlarged; adenocarcinoma of both with calcified *S. haematobium* eggs; no granuloma or fibrosis |
| 51. | Fender *et al.* | 1996 | U.K. | Prostate  Seminal vesicle | Fever, urgency, frequency, loin pain, hematuria, haemospermia, boggy prostate and seminal vesicle |
| 52. | Ingram *et al.* | 1996 | U.K. | Testicles  Epididymis | Testicular pain, haemospermia, *S. haematobium* eggs in semen |
| 53. | Lewis *et al.* | 1996 | U.K. | Testicles | Testicular pain; 2mm white lumps in semen, *S. haematobium* eggs |
| 54. | Torresi *et al.* | 1997 | Australia | - | Thin, brown discoloured semen, with many *S. haematobium* eggs |
| 55. | Vilana *et al.* | 1997 | Spain | Prostate  Seminal vesicle | Hemospermia, perineal discomfort, calcifications, enlargement on scan |
| 56 | McKenna *et al.* | 1997 | U.K. | - | Subjective ejaculate change, low volume and viscosity (watery) |
| 57. | Davies and Hamdy^#^ | 1998 | U.K. | - | Subjective change in ejaculate |
| 58. | Soans and Abel | 1999 | Australia | Right testis | 2cm mass, hypoechoic lesions; necrotizing granuloma with eggs |
| 59. | Basilio-de-Oliveira *et al.* | 2002 | Brazil | Prostate | Small nodule; adenocarcinoma with scattered *S. mansoni* eggs |
| 60. | Schwartz *et al.* | 2002 | Israel | - | Hematospermia, hematuria, pain after ejaculation, eggs in semen |
| 61. | Durand *et al.* | 2004 | France | Spermatic cord | Pain at coitus, hematuria, many *S. haematobium* eggs in semen |
| 62. | Alves *et al.* | 2004 | Brazil | Left epididymis | Chronic pain, hardening; chronic granulomatous process with eggs |
| 63. | Mortati Neto *et al.* | 2004 | Brazil | Right testis | 2cm solid nodule; granulomatous lesion with schistosome egg. |
| 64. | Faucher *et al.* | 2004 | France | Seminal vesicles  Ejaculatory duct | Hematuria, dysuria, ejaculatory pain; hyperechoic vesicles and ducts, bladder biopsies with eggs |
| 65. | Alonso *et al.* ^ǂ^ | 2006 | Spain | Testis | Dysuria, discomfort; pain on coitus; brownish, watery semen; eggs in semen |
| 66. | Lambertucci *et al.* | 2006 | Brazil | Prostate | Elevated PSA, characteristic granuloma around *S. mansoni* eggs |
| 67. | Dauda and Rafindadi | 2006 | Nigeria | Left testicle | Painless swelling, *S. haematobium* eggs viable and dead, granulomata |
| 68. | Perignon *et al.* | 2007 | France | - | Yellow coloured ejaculate, reduced in viscosity; semen *S. haematobium* eggs |
| 69. | Lopes *et al.* | 2007 | Brazil | Seminal vesicle | Elevated PSA and adenocarcinoma of the prostatic; unviable eggs in s. vesicle |
| 70. | van Delft *et al.* | 2007 | Netherlands | - | Cough, wheezing, scrotal pain, watery semen, haematospermia; eggs in semen |
| 71. | Lopes *et al.* | 2007 | Brazil | Right testis | 2cm nodule, hypoechoic; *S. mansoni* eggs with granuloma formation |
| 72. | Bacelar *et al.* | 2007 | Brazil | Prostate | Prostatic adenocarcinoma; isolated lesions, viable eggs and granulomas |
| 73. | Pawel *et al.* | 2008 | U.S.A. | Right scrotum | Pain, swelling; hydrocele; intense egg infiltrate, eosinophils; granulomas |
| 74. | Athanazio and Athanazio^#^ | 2008 | Brazil | Testicles | Massive calcified egg load in right testis; left testis only sparse calcified eggs |
| 75. | Guirassay *et al.* ^ǂ^ | 2008 | Guinea | Prostate | Pollakiuria, dysuria, terminal hematuria; enlarged prostate, fibrous with eggs |
| 76. | Kini *et al.* | 2009 | U.K. | Testicles | Primary infertility; azoospermia, reduced size, normal testes morphology |
| 77. | Lambertucci and Lippi | 2010 | Brazil | - | Few matured eggs in semen after vasectomy; none in stool, rectal biopsy |
| 78. | Stevens *et al.* | 2010 | U.K. | Rectum | Hemospermia; rectal bleeding, chronic inflammation with eggs |
| 79. | Al-Qahtani and Droupy | 2010 | France | Right testis | Primary infertility; azoospermia, white cells; small mass with eggs, granuloma |
| 80. | Rambau *et al.* | 2011 | Tanzania | Left scrotum | Pain, testicular mass; hydrocele, atrophic granulomas with schistosome eggs |
| 81. | Periyasamy *et al.* | 2011 | Malaysia | Left testicle | Genital discomfort; firm nodular swelling; eggs surrounded by granulomatous tissue |
| 82. | Hassan *et al.* | 2011 | Egypt | Right testis | Painless large tense swelling; pus drained out; many eggs with granuloma reactions |
| 83. | Hawary *et al.* | 2012 | U.K. | - | Yellow watery semen, particles; beaded seminal vesicles; eggs in urine, semen |
| 84. | Knapper *et al.* | 2012 | U.K. | - | Orange-coloured watery semen; no eggs in urine or semen; ELISA positive |
| 85. | Adisa *et al.* | 2012 | Nigeria | Left testicle | Erectile dysfunction, oligospermia; eggs of *S. mansoni* and lymphocytes, left testis |
| 86. | Kato-Hayashi *et al.* | 2013 | Japan | - | Haematuria, dysuria, hematospermia; eggs in urine and semen |
| 87. | Yu *et al.* | 2013 | China | Prostate | Frequent micturition; firm, enlarged prostate; with hyperplasia and eggs |
| 88. | Ehsani and Osunkoya | 2013 | U.S.A. | Prostate | Haemospermia, elevated PSA levels; prostate needle biopsy showed *S. haematobium* eggs, no cancer |
| 89. | Ze Ondo *et al.* ^ǂ^ | 2014 | Senegal | Testicles | 2 cases in 5 years of testicular nodule; orchidectomy done, histological analysis showed *S. haematobium* eggs |
| 90. | Sharma *et al.* ^ǂ^ | 2015 | India | Prostate | Unable to urinate, enlarged prostate with inflammatory infiltrate with eggs |
| 91. | Wobser *et al.* | 2015 | Germany | Right scrotum | Indurated subcutaneous nodule; granulomas with central necrotic areas |
| 92. | Ekenze *et al.* | 2015 | Nigeria | Left testicle | Irregular hard mass; non-caseating granulomas on viable and calcified eggs |
| 93. | Ferreira *et al* | 2015 | Brazil | Right testicle | Difficulties in urinating, hardened nodule, hypoechoic; chronic granuloma reaction |
| 94. | Alves *et al.* | 2017 | Brazil | Right testicle | Orchiepididymitis; extensive loss of testicular structure and *Schistosoma* egg-induced granulomas, plus Zika virus |
| 95. | Lang *et al.* | 2017 | Canada | Seminal vesicle | Painful ejaculation, hematospermia; cystic dilated left s. vesicle; ova in semen |
| 96. | Aber-Naser *et al.* | 2018 | Egypt | Genital organs especially both testis | Persistent azoospermia with low semen volume, absent fructose & seminal vesicles, intact spermatogenesis; *S. haematobium* & *S. mansoni* eggs in right testicular biopsy, none in the left |

| **Editorial articles** | | | |
| --- | --- | --- | --- |
| **No.** | **Authors** | **Year** | **Key facts** |
| 1. | Feldmeier *et al.* | 1999 | Male genital schistosomiasis and haemospermia |
| 2. | Murdoch | 2003 | Hematospermia associated with male genital schistosomiasis among travellers |

| **Systematic reviews** | | | |
| --- | --- | --- | --- |
| **No.** | **Authors** | **Year** | **Topics** |
| 1. | Mbabazi *et al.* | 2011 | Examining the relationship between urogenital schistosomiasis and HIV Infection. MGS and FGS implications in endemic areas. |
| 2. | Figueiredo *et al.* | 2015 | Prostate adenocarcinoma associated with prostatic  infection due to *S. haematobium*. Causal or incidental finding |
| 3. | Stecher *et al.* | 2015 | Considering treatment of male genital schistosomiasis as a tool  for future HIV prevention. More clinical studies to be conducted |

| **Literature reviews** (^ǂ^ those from references) | | | | |
| --- | --- | --- | --- | --- |
| **No.** | **Authors** | **Year** | **Organs / Specimens** | **Key facts** |
| 1. | Madden^ǂ^ | 1909 | Penis | Bilharziosis of glans, prepuce, body, erectile and subcutaneous tissues |
| 2. | Nozais *et al.* | 1983 | All genital organs | Bilharzia of seminal vesicles, prostate, testicles, epididymis |
| 3. | Al-Ghorab^ǂ^ | 1968 | Prostate  Seminal vesicles | Radiological manifestations of Genito-urinary bilharziasis |
| 4. | Richter | 2000 | - | Ultrasonographic changes on schistosomal pathologies after therapy and exposure |
| 5. | Scrimengeour and Daar | 2000 | - | Schistosomiasis review with relevance to surgeons in Australasia, includes epididymitis, orchitis, prostatitis |
| 6. | Bichler *et al.* ^ǂ^ | 2001 | - | Critical review of diagnostics and treatment of schistosomiasis, with mention of MGS |
| 7. | Corachan^ǂ^ | 2002 | - | Manifestations of schistosomiasis acquired during international travel including MGS |
| 8. | Ghoneim | 2002 | - | Bilharzial manifestations of seminal vesiculitis and prostatitis |
| 9. | Richens | 2004 | All genital organs | Genital manifestations of schistosomiasis which include bleeding and egg deposition in semen, calcifications of prostate and vesicles |
| 10. | de Cassio Saito *et al.* | 2004 | Scrotum | Ultrasound of scrotum with schistosomiasis show enlarged testes with hypoechoic solid masses (granuloma) of increased vascularity |
| 11. | Vennervald and Dunne | 2004 | - | An update on the morbidity of schistosomiasis with description of genital manifestations |
| 12. | Coon | 2005 | - | Detailed history and life-cycle of schistosomiasis including aspects of MGS |
| 13. | Maranya *et al.* | 2007 | Prostate  Seminal vesicles | Bilharzial seminal vesiculitis and prostatitis associated with haemospermia, painful ejaculation, low back pain and calcifications |
| 14. | Rollinson | 2009 | - | Overview of the history, life-cycle and clinic-pathological manifestations of schistosomiasis including MGS and its health implications |
| 15. | Shebel *et al.* | 2012 | Prostate  Seminal vesicles | Pathological radiographic findings in Genitourinary schistosomiasis |
| 16. | Le and Hsieh | 2017 | - | Review of current diagnostics and their challenges, with future advances in progress |
| 17. | Aber-Naser *et al.* | 2018 | Genital system, especially testis | Review of schistosomiasis as an important, but rarely reported cause of male infertility in endemic areas |
| 18. | Mcmanus *et al.* | 2018 | - | Review of the current epidemiology, pathophysiology, diagnosis, management and control of schistosomiasis |
